# Long-term effectiveness of a disease management program to prevent diabetic nephropathy: a propensity score matching analysis using administrative data in Japan

**DOI:** 10.1186/s12902-022-01040-4

**Published:** 2022-05-20

**Authors:** Hirohito Watanabe, Hisataka Anezaki, Kana Kazawa, Yuya Tamaki, Hideki Hashimoto, Michiko Moriyama

**Affiliations:** 1 Chronic Care and Family Nursing, Division of Nursing Science, Graduate School of Biomedical and Health Sciences, Hiroshima University, Kasumi 1-2-3, Minami-Ku, Hiroshima, 734-8553 Japan; 2 Group Business Development Division, Hankyu Hanshin Holdings Inc, Osaka, Japan; 3 Department of Health and Social Behavior, School of Public Health, The University of Tokyo, Tokyo, Japan

**Keywords:** Diabetic nephropathy, Health outcomes, Self-management, Disease management, Electronic claims database

## Abstract

**Background:**

Existing reviews indicated that disease management for patients with diabetes may be effective in achieving better health outcomes with less resource utilization in the short term. However, the long-term results were inconsistent because of the heterogeneous nature of the study designs. In the present study, we evaluated the 5-year follow-up results of a local disease management program focused on diabetic nephropathy prevention under the universal public health insurance scheme in Japan.

**Methods:**

Patients diagnosed with type 2 diabetes who had stage 3 or 4 diabetic kidney disease and were aged between 20 and 75 years were invited to join a disease management program to support self-management and receive a recommended treatment protocol between 2011 and 2013. Follow-up data were collected from an electronic claims database for the public insurance scheme. Considering the non-random selection process, we prepared two control groups matched by estimated propensity scores to compare the incidence of diabetes-related complications, death, and resource utilization.

**Results:**

The treatment group was more likely to receive clinical management in accordance with the guideline-recommended medication. After propensity score matching, the treatment group had lower incidence of diabetic nephropathy and emergency care use than the control group selected from a beneficiary pool mainly under primary care. Comparisons between the treatment group and the control group with more selected clinical conditions did not show differences in the incidence rate and resource utilization.

**Conclusions:**

The present results demonstrated limited effectiveness of the program for reducing complication incidence and resource utilization during the 5-year follow-up. Further research on the long-term effectiveness of co-management by primary care physicians, subspecialists in endocrinology and nephrology, and nurse educators is required for effective management of diabetes-related nephropathy.

## Background

The increasing prevalence of diabetes mellitus has become a significant global health policy issue because of its impact on population disease burden and related healthcare cost [1–3]. In particular, diabetic kidney disease, a frequent complication of diabetes, can lead to end-stage renal disease that requires extremely high-cost treatment and reduces the quality of life of affected patients [4, 5].

To respond to the demand for financial efficiency and improved prognosis with better quality of life, disease management has been implemented as a promising program for this purpose [6, 7]. This evidence-based approach emphasizes comprehensive care integrated across healthcare delivery systems along the continuum of the disease trajectory [8]. Disease management includes patient education, especially self-management, consecutive data monitoring, and care coordination with multi-disciplinary health professionals.

Although several systematic reviews indicated that disease management for patients with diabetes may be effective in achieving better health outcomes (e.g., reduced morbidity and disability), more appropriate processes (e.g., adherence to guidelines), less utilization of health services (e.g., hospitalization), and improved quality of life, the results remain controversial because of the heterogeneous nature of the study designs [6–10].

A long-term evaluation study in Hong Kong found a reduction in diabetes-related events, but little improvement in laboratory data for patients with severe comorbidities [11]. Another study reported a reduced incidence of stroke among patients with diabetes after an 8-year follow-up, but suffered from a low follow-up rate [12]. The study also failed to exhibit effects for other diabetes-related complications. Finally, the cost efficiency of the disease program for diabetes control was inconclusive.

In previous studies, we took advantage of the universal public health insurance scheme in Japan to overcome attrition and data quality limitations through the use of electronic administrative records [13–15]. In preliminary analyses, we found improvement of hemoglobin A1c, maintenance of renal function, and modification of patient behavior during a mean 1-year follow-up [13, 14]. We also found reduced cost during a mean 2-year follow-up that was attributable to amended treatment processes and improved prognosis of the disease [15]. However, the majority of the previous studies including ours had a short-term design, with an evaluation period of less than 3 years.

We believe that evaluation of the long-term clinical effectiveness and cost efficiency of this type of disease management program is critically important for diabetes control because of the long-term impact on patient health and healthcare cost. The present study aimed to examine the long-term effects of a local disease management program focused on diabetic nephropathy prevention using administrative data under the universal public health insurance scheme in Japan to extend our previous findings.

## Methods

### Study setting and disease management program

This study took advantage of a public health insurance scheme, the National Community-based Health Insurance System in Japan. The National Community-based Health Insurance System is a mandatory public health insurance scheme for local self-employed residents, retired citizens, and their dependents. The insurance provides universal coverage of outpatient, inpatient, dental, and prescription services with 10%–30% copayment under a monthly upper limit charge [16].

More specifically, the present study used a local disease management program setting driven by the local public health insurance authority for Kure City, a large city in West Japan with a population of about 240,000 people. The insurance authority obtains administrative claim data linked by unique encrypted IDs containing detailed information on comorbidity diagnoses, prescription contents and interventional treatments provided, physical and cognitive functional statuses, and prognosis in electronic standardized record form [17, 18]. Using this information, the authority relied on a private information company to automatically screen candidate outpatients for the program with specific targeting of those with stage 3 or 4 diabetic kidney disease [19]. The company basically screened for outpatients who were diagnosed with type 2 diabetes and aged between 20 and 75 years. Because laboratory data were lacking, the screening relied on patterns of medication and treatment to assess clinical severity, the details of which were not made public. Patients with the following conditions were excluded: type 1 diabetes, renal replacement therapy, dementia, mental disease, terminal condition, chemotherapy, radiotherapy, severe hearing loss, intractable diseases, and certificated for long-term social care.

The list of candidate outpatients was shared with local medical care providers. The final decision on whether to invite patients to join the program was made by the attending physicians, based on their clinical evaluation of the disease stage and program feasibility. Once patients were invited and agreed to join the program, specially trained nurses provided a self-management educational program via telephone and in person to support the patients in acquiring self-management skills and making behavioral changes based on self-efficacy and an existing theoretical frame, [20–22] and conducted data monitoring in collaboration with the physicians for 6 months. Patient education was conducted by face-to-face interviews every 2 weeks for the first 2 months and by telephone every month from the third to sixth months. The nurses provided knowledge about self-management including diet, exercise, medication, stress management, and self-monitoring to the patients, and encouraged their practice. They also consulted with the patients to assess their health condition and risk factors and to make shared decisions for tailored action plans, in accordance with clinical practice guidelines and advice from endocrinologists and nephrologists as needed. During the period of 2011 to 2013, nearly 2,700 beneficiary outpatients were selected as program candidates, of whom 159 patients joined and completed the program. Among them, 5-year follow-up data were available for 153 patients.

### Study design

**Fig. 1:**
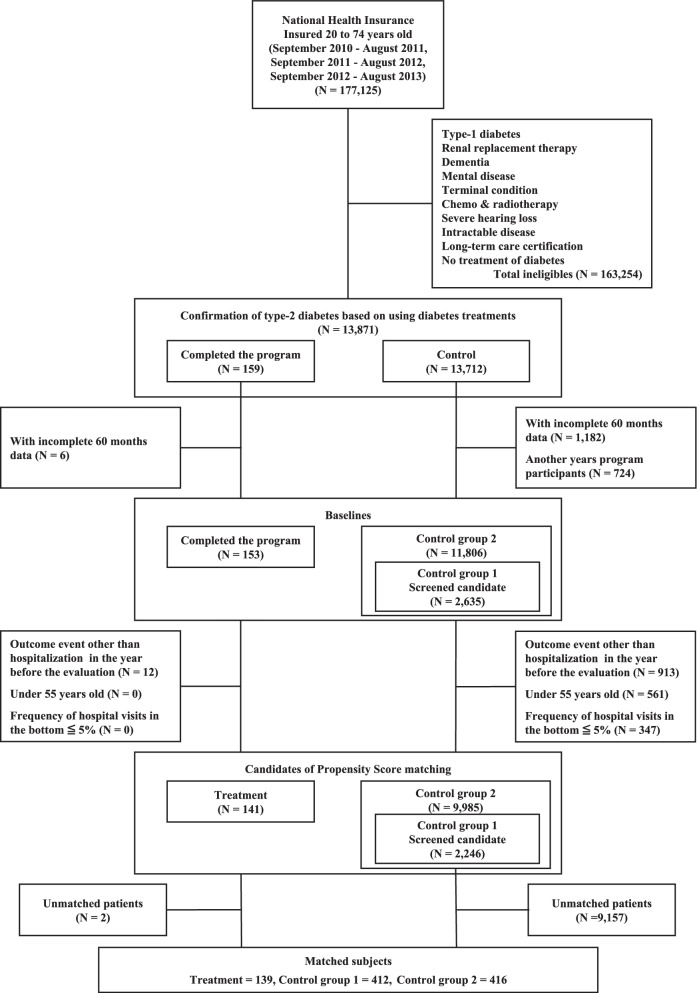
Flow chart for selection of the study subjects. Note: The number of samples and patients are described by person-years, as matching was done by multiple years

Given the non-random selection process described above, we chose to use propensity score matching to treat the selection bias when evaluating the program effectiveness. We prepared two subpopulations for the selection of control groups for this purpose. The subpopulation for control group 1 (screened candidates for program) was selected by reference to a list of candidates screened by a preset algorithm (*N* = 2,635). The subpopulation for control group 2 (beneficiaries meeting inclusion criteria) was selected from the whole beneficiary pool in Kure City who had a diagnosis of type 2 diabetes, regardless of the severity of nephropathy, were aged between 20 and 75 years, and did not have the exclusion criteria described above (*N* = 11,806) (Fig. 1).

Although we intended to equalize the clinical and other background characteristics related to the selection decision between the patients undergoing the program and the control patients, we had limited access to the full information for the automatic screening and the final decision process by the primary physicians. Consequently, we referred to the detailed information available in the claims data to reflect the patient disease stages and physician practice styles for the propensity score calculation. More precisely, we identified the use patterns for pharmaceutical prescriptions recommended in the existing clinical guidelines for the treatment of diabetes and related cardiovascular complications [23, 24], and the utilization patterns of outpatient and inpatient services (frequency of physician visits and days of in-hospital service use per year) covered by the universal public health insurance scheme.

The prescription list included oral diabetes medications (biguanides, sulfonylureas, thiazolidinediones, sodium-glucose transporter 2 inhibitors, meglitinides, dipeptidyl peptidase-4 inhibitors, alpha-glucosidase inhibitors), insulin treatment agents of any type, medications for cardiovascular risk control (e.g. anti-platelet, anti-hyperlipidemic, and anti-hypertensive agents of any kind), and cardiorenal protection medications such as angiotensin-converting enzyme and angiotensin II type 1 receptor blockers.

Propensity scores were calculated by logistic regression for participation in the disease management program regressed on age, sex, prescription patterns, comorbidities measured in the Charlson comorbidity index [25], and annual numbers of medical care utilization, outpatient visits, and hospitalization days over the past year from the baseline date. We matched the treatment and control groups for each entry year. We performed balanced 1:3 matching using the nearest-neighbor approach with replacement and a caliper width of 0.2 of the pooled standard deviation of the logit of the propensity score.

### Outcome measures

The major endpoints were occurrence of macroangiopathy (ischemic heart disease, heart failure, stroke, and other cardiovascular disease such as cerebral aneurysm and chronic peripheral arterial disease) and microangiopathy (diabetic retinopathy, neuropathy, and nephropathy) within 5 years of follow-up. The identification of these event diseases relied on diagnosis-related therapeutic medication/device use listed in the claims data to avoid misclassification due to upcoding.

Other endpoints included all-cause mortality, all-cause hospitalization, and intensive and emergency care use. We also included dependent living conditions in daily activities such as toileting, bathing, clothing, and eating as evaluated in the eligibility criteria for long-term care insurance [26]. We considered that eligibility level of ≥ 2 indicated loss of independence in daily life activities.

Finally, the utilization of medical and long-term care services was evaluated by referring to the reference prices set in the standardized item-by-item fee schedule in the Japanese universal public health insurance scheme. All costs were expressed in US dollars (USD) with an exchange rate of 1 USD = 108 Japanese yen.

### Statistical analysis

Descriptive statistics were compared between the treatment and control groups before and after propensity score matching. Unmatched patients based on common support of the propensity scores were excluded from the analysis. Standardized differences were evaluated to confirm the effectiveness of the balancing. Next, a Cox proportional hazard model was used to account for time-to-event with censoring. Multivariate analyses were adjusted for age, sex, comorbidities, and use of oral diabetes medication and/or insulin. We regarded the major endpoints and all-cause death as competing risks, and treated observations as censored at the time when a competing event occurred.

Finally, we compared medical and long-term care cost, number of outpatient visits, and number of days of hospitalization using a *t*-test and log-transformed variables.

All analyses were performed using STATA version 15 software (Stata Corporation, College Station, TX, USA).

## Results

Table 1 shows the baseline characteristics of the treatment group and the subpopulations for the two control groups before propensity score matching.

**Table 1:** Baseline characteristics of the treatment group and the subpopulations for the two control groups

**Characteristics**	**Treatment**		**Control group 1 Screened candidate**		**Control group 2 Beneficiary within inclusion criteria**	
	**n = **	153	**n = **	2,635	**n = **	11,806
Age (mean (SD)) (years)	68.0	(3.9)	67.2	(5.7)	67.1	(6.6)
Sex (male %)	90	(58.8)	1,174	(44.6)	5,284	(44.8)
Diabetes treatment medication (n, %)						
Oral	114	(74.5)	2,062	(78.3)	10,080	(85.4)
Insulin	39	(25.5)	573	(21.7)	1,726	(14.6)
Medication for cardiovascular risk control (n, %)						
Anti-platelet	43	(28.1)	631	(23.9)	2,657	(22.5)
Anti-hyperlipidemic	92	(60.1)	1,500	(56.9)	6,370	(54.0)
Anti-hypertensives	111	(72.5)	1,769	(67.1)	7,450	(62.3)
Cardiorenal protective agents (n, %)	95	(62.1)	1,397	(53.0)	5,669	(48.0)
Charlson Risk Index (n, %)						
1	119	(77.8)	1,976	(75.0)	8,614	(73.0)
2	30	(19.6)	563	(21.4)	2,682	(22.7)
3 or more	4	(2.6)	96	(3.6)	510	(4.3)
**Utilization patterns (annual) (mean (SD))**						
Medical cost (USD)	5,695	(4,855)	5,869	(7,651)	5,286	(7,710)
Number of Physician visits	33.4	(37.0)	31.1	(34.7)	29.3	(34.1)
Hospitalization days	3.7	(12.8)	3.9	(14.3)	3.8	(14.8)

The patients in the treatment group were more likely to be male, receive treatment with insulin, and have medication for cardiovascular risk control and cardiorenal protection, and had a lower Charlson comorbidity index than the patients in the two subpopulations for the control groups. The treatment group had more frequent physician visits, fewer days of hospitalization, and lower medical cost utilization.

Table 2 presents the comparisons between the treatment group and the control groups after propensity score matching.

**Table 2:** Characteristics of the treatment group and the control groups after propensity score matching

Characteristics	Treatment		Control group 1 Screened candidate		Control group 2 Beneficiary within inclusion criteria		Standardized difference treatment—control group1	Standardized difference treatment—control group2
	N =	139	N =	412	N =	416		
Age (mean (SD)) (years)	68.1	(3.8)	67.9	(4.0)	68.5	(4.1)	0.051	-0.101
Sex (male %)	83	(59.7)	266	(64.6)	246	(59.1)	-0.100	0.012
Diabetes treatment medication (n, %)								
Oral	105	(75.5)	295	(71.6)	321	(77.2)	0.089	-0.038
Insulin	34	(24.5)	117	(28.4)	95	(22.8)	-0.089	0.038
Medication for cardiovascular risk control (n, %)								
Anti-platelet	38	(27.3)	113	(27.4)	126	(30.3)	-0.002	-0.065
Anti-hyperlipidemic	85	(61.2)	258	(62.6)	272	(65.4)	-0.030	-0.088
Anti-hypertensives	102	(73.4)	298	(72.3)	301	(73.4)	0.024	0.023
Cardiorenal protective agents (n, %)	89	(64.0)	263	(63.8)	263	(63.2)	0.004	0.017
Charlson Risk Index (n, %)								
1	114	(82.0)	334	(81.1)	327	(78.6)	0.024	0.086
2	22	(15.8)	66	(16.0)	81	(19.5)	-0.005	-0.096
3 or more	3	(2.2)	12	(2.9)	8	(1.9)	-0.048	0.017
Utilization patterns (annual) (mean (SD))								
Medical cost (USD)	4,991	(3,871)	5,014	(5,614)	5,808	(5,741)	-0.005	-0.167
Number of Physician visits	32.4	(37.1)	30.4	(33.5)	35.2	(38.5)	0.057	-0.074
Hospitalization days	2.4	(9.9)	2.0	(10.6)	2.0	(8.0)	0.039	0.044

After the propensity score matching, the differences in demographic characteristics and prescription patterns largely disappeared, with standardized differences below 0.1. The number of physician visits and annual medical cost were higher in control group 2 (beneficiaries meeting clinical criteria) compared with the treatment group and control group 1 (screened candidates for program).

Table 3 shows the cumulative incidence of targeted events during the 5-year follow-up. Control group 2 had higher incidence of diabetes-related complications of any kind (20.4% vs. 12.9%), nephropathy requiring hemodialysis (4.3% vs. 0.7%), and emergency care use (25.5% vs. 15.1%) than the treatment group. There were no marked differences in incidence between the treatment group and control group 1.

**Table 3:** Cumulative incidence of targeted events during the 5-year follow-up

	Treatment	Control group 1 Screened candidate	Control group 2 Beneficiary within inclusion criteria
Diabetes-related complications (all)	12.9%	13.3%	20.4%*
Ischemic heart disease	3.6%	2.9%	2.2%
Stroke	0.7%	1.5%	3.1%
Retinopathy requiring surgery	4.3%	5.1%	6.3%
Neuropathy	3.6%	1.5%	2.4%
End-stage renal disease requiring dialysis	0.7%	1.2%	4.3%*
Intensive care use	5.0%	5.3%	8.4%
Emergency care use	15.1%	18.4%	25.5%**
All-cause hospitalization	51.8%	56.1%	59.4%
Dependency in activities of daily living	2.2%	2.7%	4.3%
All-cause mortality	3.6%	2.9%	2.2%

^*^P < 0.05 **P < 0.01 for Fisher's exact test compared with treatment group

Table 4 presents the results of the Cox proportional hazard model analyses on event incidence during the 5-year follow-up, adjusted for age, sex, diabetes medication, and Charlson comorbidity index. None of the estimated hazard ratios reached conventional significance levels between the treatment group and control group 1 (screened candidates for program). However, the treatment group had lower hazard ratios for diabetes-related complication of any kind and emergency care use than control group 2 (beneficiaries meeting inclusion criteria).

**Table 4:** Estimated hazard ratios of event incidence in the treatment group compared to control groups

	vs. Control group 1 Screened candidate		vs. Control group 2 Beneficiary within inclusion criteria	
Diabetes-related complications (all)	1.14	(0.67–1.93)	0.60*	(0.36–1.00)
Ischemic heart disease	1.38	(0.48–3.93)	1.67	(0.56–5.02)
Stroke	0.43	(0.05–3.67)	0.21	(0.03–1.59)
Retinopathy requiring surgery	1.25	(0.53–2.97)	0.65	(0.27–1.59)
Neuropathy	2.71	(0.82–8.92)	1.49	(0.51–4.37)
End-stage renal disease requiring dialysis	0.63	(0.07–5.52)	0.15	(0.02–1.16)
Intensive care use	1.09	(0.48–2.45)	0.63	(0.28–1.43)
Emergency care use	0.79	(0.49–1.29)	0.59*	(0.37–0.94)
All-cause hospitalization	0.90	(0.69–1.18)	0.82	(0.63–1.06)
Dependency in activities of daily living	0.78	(0.22–2.80)	0.69	(0.20–2.38)
All-cause mortality	1.50	(0.51–4.41)	0.72	(0.27–1.90)

^*^P < 0.05

Adjusted for age, sex, diabetes medication, and Charlson comorbidity index

Finally, we compared the medical and long-term care cost among the groups for the 5-year follow-up period. The mean medical and long-term care cost for the treatment group was 34,836 USD (90% confidence interval [CI]: 29,865–39,807 USD), compared with 37,758 USD (90% CI: 34,354–41,161) in control group 1 (screened candidates for program) and 45,336 USD (90% CI: 41,152–49,519 USD) in control group 2 (beneficiaries meeting inclusion criteria).

## Discussion

This study investigated the long-term effects of a disease management program for diabetic nephropathy prevention among high-risk outpatients diagnosed with diabetes covered by a local public health insurance system in Japan. Contrary to our earlier findings on the short-term effects of the program [14–16], the present results provided only limited evidence to support the effectiveness of the program for risk reduction of major clinical events and medical care utilization during a 5-year follow-up period.

One possible explanation for the non-positive findings is that the program may delay the onset of clinical events for a short time, but its long-term effectiveness may be attenuated by other competing factors such as physical aging, increased risk of chronic conditions related to aging, and secular trends that equally affect treated and untreated patients over several years. Likewise, despite the encouraging preventive effects observed in an earlier phase, the recent evaluation in the Diabetes Prevention Program Outcomes Study revealed null results after 15 years of follow-up in terms of microvascular complication incidence between the treatment group and the control group [27].

Another possible explanation is that changes in practice styles following revision of the practice guidelines for diabetic nephropathy during the study period may have contaminated the program outcomes. Compared with the 2013 practice guidelines for chronic kidney disease management, the 2018 revised guidelines adopted evolving practice recommendations based on newly available evidence that were specifically related to the management of diabetes-related nephrology [24]. Improved clinical practice patterns, even in the control groups, may have nullified the effectiveness of the intervention program. Re-evaluation with patients under the recent practice guideline recommendations may be necessary to confirm the current null findings.

In the present study, treatment assignment was performed at the patient level. Therefore, the fact that several patients were under medical care by identical physicians may have contaminated the interventional effect through a physician response bias upon awareness of being observed. This response bias could certainly explain the null results between the treatment group and control group 1, both of whom were included in the screening candidate list presented to the physicians.

We also speculate that the current program may have failed to select the most suitable subpopulation of high-risk patients for preventive interventions against diabetic nephropathy. A recent review indicated that diabetic nephropathy is a heterogeneous clinical category that requires sophisticated risk segmentation for personalized treatment to achieve effective prevention [5, 28, 29]. Unspecified heterogeneous background differences between the treated and untreated patients, if they exist, may have confounded the results.

When control group 2 (beneficiaries meeting inclusion criteria) was compared with the other two groups, the baseline characteristics indicated that these patients received poorer quality of care for effective diabetes treatment, in that their prescription rate for cardiorenal protection medication was lower than the rates in the other groups. The patients in the beneficiary pool population were most likely under medical care by primary care practitioners who may not be experts in diabetes and nephrological subspecialty. The patients in the treatment group and control group 1, who were most likely under supervision by subspecialists, had lower incidence of diabetes-related complications, specifically end-stage nephropathy, and lower utilization of emergency care. A previous study in Japan revealed suboptimal clinical practice for diabetes control in general to effectively detect nephropathy and retinopathy [30]. The current program may be strengthened by facilitating co-management with primary care physicians and subspecialists in endocrinology and nephrology to improve the effectiveness of the preventive interventions against diabetic nephropathy [31].

As we discussed, our non-positive finding about an expected effect of the disease management program may have arisen for several reasons: limited effectiveness of the disease management program per se, poor choice of the control group for efficient comparison, or contamination by non-observed influential factors over the long period of 5-year follow-up. Unfortunately, the currently available data do not allow us to determine the main reason for the null finding. Future research should consider a suitable design to overcome these factors for more efficient evaluation of the program effectiveness. At least, our finding in favor of patients under supervision by subspecialists after screening processes would indicate disease management by timely monitoring of administrative data may be beneficial to support patients with diabetes to control diabetic nephropathy.

Although strengths of the present study can be found in the small attrition and data quality in an electronic claims database under the universal public health insurance scheme, we should mention several limitations of the study. First, due to the non-randomized design, remaining unobserved confounders may have affected the results. Second, we did not examine patient socioeconomic factors such as living environment and family support, which may have been influential on the outcomes. Third, we did not include indirect costs such as lost productivity of the patients. Fourth, the lack of available laboratory data, including HbA1c, in the study may have led to misclassification of the disease severity, which in turn may have resulted in underestimation of the effect of the disease management program. Finally, a larger size of program inclusion with a more comparative design would have allowed a better statistical power to detect the effectiveness of the disease management program, which must await further research in the future.

## Conclusions

To conclude, taking advantage of the universal public health insurance scheme in Japan, we revealed the 5-year follow-up results of a disease management program targeting diabetic nephropathy prevention. After propensity score matching, the results demonstrated limited effectiveness of the program for reducing complication incidence and resource utilization. Further research is warranted on the long-term effectiveness of co-management by primary care physicians, subspecialists in endocrinology and nephrology, and nurse educators for prevention of diabetes-related nephropathy.

## Data Availability

Data use is strictly limited to those with official approval from the Kure City National Health Insurance authority. Data for this study are deidentified data provided by the authority, and are not publicly available. Inquiries for data contents should be directed to the corresponding author and may be available subject to appropriate approvals from the Kure City National Health Insurance authority.
